# Haloperidol induced Pisa syndrome in a patient with treatment resistant schizophrenia

**DOI:** 10.1192/j.eurpsy.2024.1579

**Published:** 2024-08-27

**Authors:** I. Yaich, A. Touiti, C. Ben Said, N. Bram

**Affiliations:** ^1^Forensic Psychiatry Departement, Razi Hospital, La Manouba; ^2^Faculty of Medecine of Tunis, Tunis El Manar University, Tunis, Tunisia

## Abstract

**Introduction:**

Acute dystonia, an adverse effect of neuroleptics, is linked to D2 neuronal receptor hypersensitivity or neurotoxicity due to oxidative stress mechanisms. Pisa syndrome (PS) or Pleurothotonus, a relatively uncommon condition, manifests as dystonia of the trunk and is potentially reversible with early intervention.

**Objectives:**

To describe PS following haloperidol decanoate injection in a treatment-resistant schizophrenia (TRS) patient, identify associated risk factors, and present therapeutic options.

**Methods:**

We provide a comprehensive case description and perform a PubMed database search using the following keywords: “Pisa syndrome,” “dystonia,” “schizophrenia,” and “antipsychotic”.

**Results:**

A 54-year-old man with TRS, previously treated with 100 mg of haloperidol decanoate and 10 mg of olanzapine due to clozapine-induced myocarditis, exhibited hallucinatory delusional syndrome and behavioral disturbances. Neurological examination, lab tests, and brain imaging confirmed a psychotic relapse. Haloperidol decanoate dosage was increased to 150 mg. Four days later, the patient developed a trunk tilt that resolved after receiving anticholinergic treatment.Despite PS being more common in females and associated with brain conditions, this patient presented multiple risk factors, including prolonged typical antipsychotic treatment, advanced age, and an increase in antipsychotic doses. Discontinuing the causative antipsychotic or adding synthetic anticholinergics led to symptom reversibility.

**Conclusions:**

PS is a rare occurrence. Understanding associated risk factors and frequently implicated medications is crucial for elucidating the phenomenon and managing the disorder

**Disclosure of Interest:**

None Declared

